# Kinome Profiling of NF1-Related MPNSTs in Response to Kinase Inhibition and Doxorubicin Reveals Therapeutic Vulnerabilities

**DOI:** 10.3390/genes11030331

**Published:** 2020-03-20

**Authors:** Jamie L. Grit, Matt G. Pridgeon, Curt J. Essenburg, Emily Wolfrum, Zachary B. Madaj, Lisa Turner, Julia Wulfkuhle, Emanuel F. Petricoin, Carrie R. Graveel, Matthew R. Steensma

**Affiliations:** 1 Center for Cancer and Cell Biology, Van Andel Research Institute, Grand Rapids, MI 49503, USA; jamie.grit@vai.edu (J.L.G.); matthew.pridgeon@helendevoschildrens.org (M.G.P.); curt.essenburg@vai.org (C.J.E.); carrie.graveel@vai.org (C.R.G.); 2 Helen DeVos Children’s Hospital, Spectrum Health System, Grand Rapids, MI 49503, USA; 3 Bioinformatics & Biostatistics Core, Van Andel Research Institute, Grand Rapids, MI 49503, USA; emily.wolfrum@vai.org (E.W.); zachary.madaj@vai.org (Z.B.M.); 4 Pathology and Biorepository Core, Van Andel Research Institute, Grand Rapids, MI 49503, USA; lisa.turner@vai.org; 5 Center for Applied Proteomics and Molecular Medicine, George Mason University, Manassas, VA 22030, USA; jwulfkuh@gmu.edu (J.W.); epetrico@gmu.edu (E.F.P.); 6 Michigan State University College of Human Medicine, Grand Rapids, MI 49503, USA

**Keywords:** MPNST, NF1, kinase, kinome adaptation, kinome reprogramming, MET, MEK, doxorubicin, capmatinib, tram

## Abstract

Neurofibromatosis Type 1 (NF1)-related Malignant Peripheral Nerve Sheath Tumors (MPNST) are highly resistant sarcomas that account for significant mortality. The mechanisms of therapy resistance are not well-understood in MPNSTs, particularly with respect to kinase inhibition strategies. In this study, we aimed to quantify the impact of both the genomic context and targeted therapy on MPNST resistance using reverse phase phosphoproteome array (RPPA) analysis. We treated tumorgrafts from three genetically engineered mouse models using MET (capmatinib) and MEK (trametinib) inhibitors and doxorubicin, and assessed phosphosignaling at 4 h, 2 days, and 21 days. Baseline kinase signaling in our mouse models recapitulated an MET-addicted state (NF1-MET), P53 mutation (NF1-P53), and HGF overexpression (NF1). Following perturbation with the drug, we observed broad and redundant kinome adaptations that extended well beyond canonical RAS/ERK or PI3K/AKT/mTOR signaling. MET and MEK inhibition were both associated with an initial inflammatory response mediated by kinases in the JAK/STAT pathway and NFkB. Growth signaling predominated at the 2-day and 21-day time points as a result of broad RTK and intracellular kinase activation. Interestingly, AXL and NFkB were strongly activated at the 2-day and 21-day time points, and tightly correlated, regardless of the treatment type or genomic context. The degree of kinome adaptation observed in innately resistant tumors was significantly less than the surviving fractions of responsive tumors that exhibited a latency period before reinitiating growth. Lastly, doxorubicin resistance was associated with kinome adaptations that strongly favored growth and survival signaling. These observations confirm that MPNSTs are capable of profound signaling plasticity in the face of kinase inhibition or DNA damaging agent administration. It is possible that by targeting AXL or NFkB, therapy resistance can be mitigated.

## 1. Introduction

Targeting the RAS/ERK signaling pathway is an effective treatment for numerous cancers with hyper-activation of the RAS pathway. The most striking clinical responses to inhibitors of BRAF, MEK, and EGFR have been observed in melanoma and lung cancers where RAS pathway activation is intrinsic. Even though these targeted therapies have resulted in an extension of the overall survival in these inherently aggressive cancers, the clinical response is often transient and complete remission is rare. Resistance to kinase inhibition is a significant clinical challenge and numerous studies have identified multifactorial and heterogeneous mechanisms of resistance to kinase inhibition [1]. 

Malignant Peripheral Nerve Sheath Tumors (MPNSTs) are aggressive, highly chemoresistant sarcomas arising from Schwann cells that are a leading cause of death in patients with Neurofibromatosis Type 1 (NF1) [2,3]. Neurofibromatosis Type 1 (NF1) is caused by germline mutations in the *NF1* gene and is the most common single-gene disorder, affecting 1 in 3000 live births. The *NF1* gene encodes neurofibromin, a GTPase-activating protein that negatively regulates RAS (including HRAS, NRAS, and KRAS), where the loss of NF1 leads to deregulated RAS signaling. Deregulated RAS signaling caused by the loss of neurofibromin is both permissive and instructive for MPNST progression (3–5). Recent clinical trials have focused on targeting members of the RAS signaling pathway or the PI3K/mTOR pathway. To date, these trials have failed to identify consistent therapeutic vulnerabilities in MPNSTs; however, few studies have examined why these therapies failed. These clinical results highlight our limited knowledge of the mechanisms that drive resistance to kinase inhibition in MPNSTs.

In addition to loss of the *NF1* gene, NF1-related MPNSTs exhibit highly complex genomic alterations that result in substantial tumor suppressor gene loss and oncogene copy number variations [4,5]. How MPNST genomic alterations affect therapy resistance is currently unclear. Recently, we performed a genomic analysis of longitudinally collected MPNST samples. This study revealed the early concomitant presence of *MET*, *HGF*, and *EGFR* amplifications, as well as the site-specific expansion of these loci over time and treatment. These data point to an adaptive mechanism involving RTK signaling for both malignant transformation and clonal selection in MPNSTs [6]. To advance our understanding of the MPNST therapeutic response and resistance to RAS pathway inhibition, we developed diverse preclinical NF1-related MPNST models, including an “MET-addicted” model of NF1-related MPNSTs (NF1-MET), an *Nf1/Trp53*-deficient model (NF1-P53), and an NF1 model (P53^WT^, *Hgf*-amplified) [7,8,9]. Using these MPNST models, we determined that P53 deficiency significantly exacerbates resistance to MEK inhibition; however, combined MEK and MET inhibition overcame therapy resistance [6]. Importantly, these results demonstrated that NF1-related MPNSTs maintain multiple signaling dependencies beyond RAS, and that genomic determinants, such as P53 and RTK genomic alterations, profoundly influence the therapy response.

Kinome reprogramming is a powerful barrier to a durable treatment response to kinase inhibition [10,11,12]. These signaling adaptations occur as a result of the compensatory activation of evolutionarily conserved signaling pathways that drive growth and proliferation, especially when central pathways such as RAS/MEK and PI3K/mTOR are blocked by drugs [13]. Kinome adaptation leads to diverse mechanisms of therapy resistance that can be classified as three categories, all of which can occur simultaneously during treatment [1]. The most common resistance mechanism is defined as “pathway reactivation”, which can occur through multiple mechanisms that reinforce oncogenic signaling in the face of strong target inhibition. The second common mechanism of resistance is “pathway bypass”, which occurs when oncogenic pathways are activated at a downstream convergence point by a parallel pathway, despite effective upstream inhibition (e.g., PI3K/AKT/ERK activation during MEK inhibition). The third resistance mechanism is “pathway indifference”, where the cancer cells transition to an alternative survival state that is independent of the targeted oncogenic pathway. Each of these resistance mechanisms have been observed in response to kinase inhibition in cancers with RAS-activation dependencies. Efforts to delineate patterns of therapy resistance have been valuable in understanding the treatment response and the identification of targets for salvage therapy. Even though genetic mechanisms of resistance (e.g., somatic mutations or gene amplifications) have been a major focus of resistance research, the impact of phosphoproteomic changes in therapeutic resistance has been increasingly acknowledged [1].

The mechanisms that regulate adaptive kinome reprogramming in NF1-deficient cancers are not well-elucidated. In this study, we aimed to define both mechanisms and categories of resistance to standard chemotherapy and targeted kinase inhibition in NF1-related MPNSTs in order to identify 1) novel therapeutic strategies that correlate with the genomic status or 2) salvage therapies that are focused on the emerging ‘resistant’ tumor populations. Kinome reprogramming can be heavily influenced by genomic alterations (i.e., amplification and mutation) of kinases, the overexpression of other kinases, or ligand activation [13,14,15,16]. Our genomic analysis of MPNST progression identified genomic events during human MPNST progression that heighten RTK signaling, AKT/mTOR activation, and cell survival [6]. To understand how these genomic contexts influence the therapy response to kinase inhibition, we conducted a phosphoproteomic analysis using a reverse phase protein array (RPPA) in our established MPNST mouse models. RPPA is a powerful research tool that simultaneously interrogates a broad range of phosphosites across the entire kinome, including activating, inactivating, and alternative sites. Using this comprehensive analytical technique, we confirmed substantial signaling heterogeneity following kinase inhibition in NF1-related MPNSTs leading to therapy resistance, including examples of pathway reactivation, bypass, and indifference. Our data verifies that a broad range of signaling pathways are activated as a result of effective MET, MEK, and MET/MEK blockade, most notably, AXL, NFκB, RAS/RAF/MEK, and AKT/mTOR pathways. Interestingly, the patterns of kinome adaptation were distinct based on the kinase target and the genomic context of the tumor. We also demonstrated that administration of the DNA damaging reagent doxorubicin resulted in a distinct pattern of kinome adaptation that was partially, but not fully, mitigated by MET and MEK inhibitors. Categorizing these patterns of therapy resistance is valuable for inferring patient stratification and the identification of targets for salvage therapy.

## 2. Materials and Methods

### 2.1. Murine MPNST Tumorgrafts and Treatment

Immediately following the euthanasia of tumor-bearing mice, 15–25 mg portions of each tumor were transplanted into the flank of NSG-SCID mice using a 10 gauge trochar. Tumors were measured twice weekly and euthanized when the tumor size exceeded 2500 mm^3^. When the tumor volume reached approximately 150 mm^3^, mice were randomized into treatment groups, treated, and euthanized as independent groups at 4-h, 2-day, or 21-day time points (or until mice reached the euthanasia criteria). Respective doses across all treatment combinations were capmatinib (30 mg/kg twice daily vial oral gavage), trametinib (1 mg/kg daily via oral gavage), and doxorubicin (1 mg/kg once via subcutaneous injection). For the 4-h time point, all animals were euthanized 4 h after a single dose. For the 2-day time point, capmatinib-treated animals received three total doses and were euthanized 4 h after the final treatment, whereas trametinib-treated animals received two total doses before euthanasia. Tumors were immediately harvested and either snap-frozen or formalin-fixed for further analysis. Three representative tumors were assessed by RPPA for each time point, treatment, and genotype group. Capmatinib and trametinib were obtained from Novartis. Doxorubicin was obtained from LC Laboratories. All animal experimentation in this study was approved by the Van Andel Institute’s Internal Animal Care and Use Committee (XPA-19-04-001).

### 2.2. Sample Collection and Preparation for RPPA Downstream Analysis

Samples were frozen in liquid nitrogen within 20 min upon surgical resection to preserve the integrity of the phosphoproteome. Specimens were then embedded in an optimal cutting temperature compound (Sakura Finetek, Torrance, CA, USA), cut into 8 μm cryo-sections, mounted on uncharged glass slides, and stored at −80 °C until microdissected. Each slide was fixed in 70% ethanol (Sigma Aldrich, Darmstadt, Germany), washed in deionized water, stained with hematoxylin (Sigma Aldrich, Darmstadt, Germany) and blued in Scott’s Tap Water substitute (Electron Microscopy Sciences), and dehydrated through an ethanol gradient (70%, 95%, and 100%) and xylene (Sigma Aldrich, Darmstadt, Germany). In order to prevent protein degradation, complete protease inhibitor cocktail tablets (Roche Applied Science, Basel, Switzerland) were added to the ethanol, water, hematoxylin, and Scott’s Tap Water substitute [17]. For each sample, an average of 15,000 tumor cells was isolated from the surrounding microenvironment using a Pixcell II LCM system (Arcturus, Mountain View, CA, USA). Microdissected cells were lysed in a 1:1 solution of 2× Tris-Glycine SDS Sample buffer (Invitrogen Life Technologies, Carlsbad, CA, USA) and Tissue Protein Extraction Reagent (Pierce, Waltham, MA, USA) supplemented with 2.5% of 2-mercaptoethanol (Sigma Aldrich, Darmstadt, Germany). Cell lysates were boiled for 8 min and stored at −80 °C.

### 2.3. Reverse Phase Protein Microarray Construction and Immunostaining

Using an Aushon 2470 arrayer (Aushon BioSystems, Billerica, MA, USA) equipped with 185 μm pins, samples and standard curves for internal quality assurance were printed in triplicate onto Oncyte Avid nitrocellulose-coated slides (Grace Bio-labs, Bend, OR, USA), as previously described [17]. A Sypro Ruby Protein Blot Stain (Molecular Probes, Eugene, OR, USA) protocol was used to stain selected arrays to quantify the total amount of protein within each sample.

Before immunostaining, each array was first incubated with Reblot Antibody stripping solution (Chemicon) for 15 min at room temperature, followed by two washes in PBS. To minimize potential nonspecific bindings, arrays were then incubated in I-block solution (Invitrogen Life Technologies, Carlsbad, CA, USA) for 1 h. Each array was tested with a single primary antibody using an automated system (Dako, Santa Clara, CA, USA). The antibody specificity was tested by immunoblotting using a wide panel of cell lysates, as previously described [17,18]. Negative control arrays were incubated with the anti-rabbit secondary antibody only to account for unspecific binding and background noise. A commercially available catalyzed signal amplification system (Dako, Santa Clara, CA, USA) coupled with a biotinylated anti-rabbit secondary antibody (Vector Laboratories) and a streptavidin-conjugated IRDye680 (LI-COR Biosciences, Lincoln, NE, USA) were used for the amplification and detection of the fluorescent signal. Arrays were probed with a total of 99 antibodies targeting protein kinases involved in major cellular functions, and the results of the broad screening were previously published.

Antibody and Sypro Ruby-stained arrays were scanned using a laser-based PowerScanner (TECAN, Mönnedorf, Switzerland). Acquired images were analysed using the MicroVigene software version 5.1 (Vigene Tech, Carlisle, MA, USA). This commercially available software performs spot finding, averages the triplicates, subtracts the background from the negative control slide(s), and normalizes each sample to the corresponding amount of total protein measured by Sypro Ruby staining. Intra- and inter-assay reproducibility of the RPPA platform has been previously reported [19,20].

### 2.4. Statistical Methods

*Tumor growth analysis*: Linear mixed-effects models, with random slopes and intercepts, and false discovery rate-adjusted contrasts, were used to estimate and compare tumor growth rates for the different mono and combo therapies. For visualization of the changes in tumor growth, the tumor volume was imputed using the last observation carried forward, until the animal was euthanized. Curves terminated once >50% of mice had been euthanized in the respective treatment group. All analyses were conducted using R v3.2.2 (https://cran.r-project.org/), with an assumed level of significance of α = 0.05.

*Proteomic analysis*: Fold change in expression for each phosphosite was calculated by log_2_ transformation of the treatment relative to the vehicle mean for that genotype group. Fold change was ranked-ordered by the median of the treatment group for each genotype and for each condition, the top and bottom 15 proteins from the ranked list were plotted in balloon plots. A total of 98 protein sites passed quality control metrics and were used for analysis. Fold change in the expression for each protein was calculated as the protein expression relative to the vehicle mean for that genotype-time group. Proteins were rank-ordered on the *y*-axis by the median transformed fold change for each treatment-genotype-time group and plotted from highest to lowest fold change. Each column represents a single animal. For the 4-h and 2-day time points, animals were plotted randomly on the x-axis. For the 21-day time point, animals were plotted on the *x*-axis based on tumor size (largest to smallest) at sacrifice, corresponding to tumors 4–6, respectively, in the associated tumor-graft plots. The balloon color indicates the log_2_ fold change in protein expression. Proteins with a greater than 4-fold increase or decrease in expression relative to the vehicle were plotted as log_2_ fold change >2 or <−2, respectively. The balloon size indicates the absolute protein expression normalized to the total protein input and background. Head-tail balloon-maps were created by plotting the 30 proteins with the highest and lowest fold change in expression for each treatment-genotype-time group. Plots were generated using R v3.6.1. For pospho-AXL and phospho-NFkB correlation analysis, normality was first assessed by a Shapiro–Wilk normality test. Correlations were analyzed by two-sided Spearman’s rank correlation rho. For correlation plots, data were fit by stat_smooth using loess with span = 1 using ggplot2 (v3.2.1). Missing data points (due to a failure to meet RPPA quality control standards) were omitted from the analysis. All analyses were done using R (v3.6.2).

## 3. Results

### 3.1. Distinct Kinome Response to MET Inhibition Present in MET-Addicted MPNSTs

**Figure 1:**
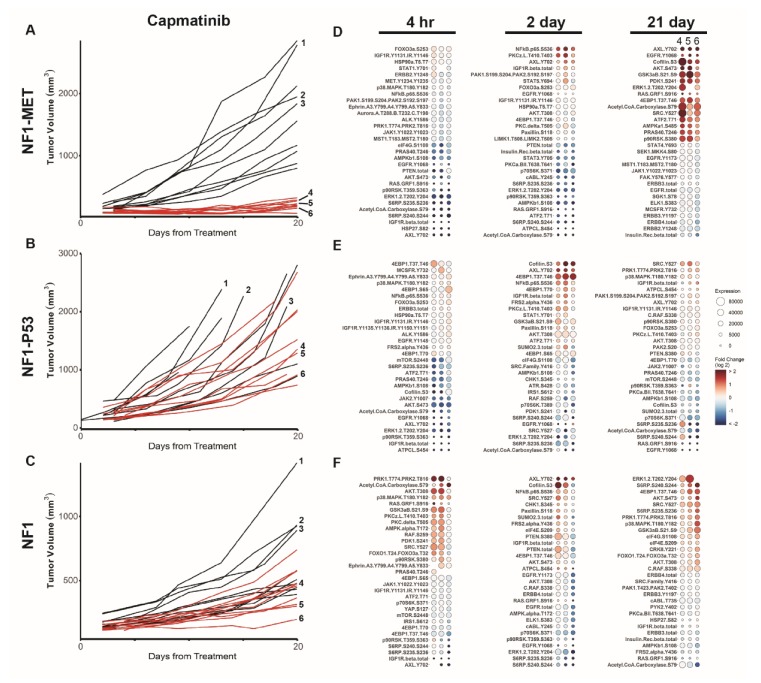
MET inhibition reveals differential innate and adaptive kinome reprogramming. Individual tumor growth curves for (**A**) Neurofibromatosis Type 1 (NF1)-MET, (**B**) NF1-P53, and (**C**) NF1 tumorgrafts plotted by treatment (colored lines) compared to the vehicle (black lines). The analysis of tumor growth data was previously reported [6]. The annotated tumors were analyzed by a reverse phase phosphoproteome array (RPPA) (**D**–**F**). The fold change relative to the mean protein expression of control tumors (i.e., #1–3) was calculated for each tumor #4–6, with the first column of Panel A at 21 days corresponding to tumor #4, the second to tumor #5, and the third to tumor #6. Ranked balloon plots of the proteins with the highest and lowest fold change in expression after 4-h, 2-day, and 21-day treatment of the NF1-MET model with capmatinib. Each column represents a single animal. Balloon color indicates the fold change in expression relative to the vehicle mean (*n* = 3) for that time point. Balloon size indicates the absolute protein expression normalized to the total protein input and background.

To understand how RTK amplification and enhanced RTK signaling impact the MPNST kinome, we assessed the influence of both the *MET* copy number and MET kinase inhibition on the drug response and resistance. Both *MET* and its ligand, hepatocyte growth factor (HGF), are implicated in NF1-related MPNST initiation and progression [21,22,23]. Previously, our genomic analysis of human MPNST progression revealed that *MET* and *HGF* copy number gains are present at the earliest stage of neurofibroma transformation and increase during metastasis and resistance [6]. Moreover, studies in other cancers have demonstrated that aberrant MET signaling can drive malignant progression in a variety of RAS-deregulated human tumors and augment the oncogenic effects of RAS activation [24,25]. To understand the impact of the MET genomic status on kinome adaptations, we evaluated the response and resistance to the potent and selective MET inhibitor capmatinib in three diverse models of NF1-related MPNSTs, including an “MET-addicted” model (NF1-MET), an *Nf1/Trp53*-deficient model (NF1-P53), and an NF1 model (P53^WT^, MET^WT^, *Hgf*-amplified). As we previously showed, NF1-MET MPNSTs were uniformly sensitive to MET inhibition, whereas a heterogeneous response to MET inhibition was observed in NF1-P53 and NF1 MPNSTs (Figure 1A–C) [6]. To characterize the kinome response to MET inhibition, we performed pathway activation mapping of 98 proteins and phosphoproteins. This was a targeted pathway activation analysis focused on actionable targets of RTK-mediated signaling, downstream PI3K-mTOR signaling, downstream RAS-ERK signaling, and motility/adhesion signaling. To assess the immediate, early, and late kinome responses to kinase inhibition, we profiled the tumor phosphoproteome after 4 h, 2 days, and 21 days of treatment. With these time points, we anticipated that both innate and acquired kinome adaptations would be observed in the various genomic backgrounds. Changes in the expression relative to the vehicle were plotted in rank order for each timepoint. For the 21-day RPPA analysis of each MPNST model, we analyzed tumors that had diverse treatment responses, while avoiding tumors that exhibited grossly anomalous growth patterns compared to the mean growth curve (see individual tumor annotations in Figure 1A–C). By including diverse tumors, we anticipated that we would detect the heterogeneity of mechanisms underlying drug resistance. For example, because the NF1-MET tumors are “*Met*-addicted”, substantial growth inhibition was present at 21 days and minimal heterogeneity in the drug response was observed (Figure 1A) This homogeneous response was not observed in the other MPNST tumor-graft lines (Figure 1B,C). Correspondingly, we observed a more homogeneous kinome response in NF1-MET tumors in comparison to the responses observed in NF1-P53 and NF1 tumors (Figure 1D–F; Figure S1).

After 4-h capmatinib treatment, we observed a striking repression of ERK, AKT, and RTK phosphorylation that corresponded to growth reduction in the NF1-MET tumors (Figure 1D). Overall, minimal kinome activation was observed at the 4-h time point in growing NF1-MET and NF1-P53 tumors (Figure 1D,E; Figure S1B,C); however, two of three NF1 tumors had phosphorylation changes in several pathways at the 4-h time point (i.e., PRK, AKT, and p38MAPK) (Figure 1F). After 2-day capmatinib treatment, we observed increased activating phosphorylation at several sites in the NF1-P53 and NF1 tumors, including AXL (Y702), cofilin (S3), and 4EBP1 (T37/T46) (Figure 1E,F; Figure S5), which is a finding that correlated with the relatively increased capmatinib resistance at 21 days (Figure 1B,C). In the NF1-MET tumors, NFκB demonstrated the strongest increase in phosphorylation at the 2-day time point. This probe corresponds to S536 in the transactivation domain (TAD) of NFκB/p65, which leads to transactivation. Interestingly, at the 2-day time point, NFκB/p65 was in the top three most increased phoshposites in all of the tumor models after 2-day MET inhibition. Since NFκB is a master regulator of the inflammatory response, survival, and tumor proliferation [26], and a known mediator of pathway indifference [27], NFκB activation at the 48-h time point may represent a common kinome adaptation that is agnostic to the MPNST genomic context.

After 21 days of capmatinib treatment, significant tumor death was observed in the NF1-MET tumors (Figure 1A), with only a small layer of viable cells present at the edge of the tumors [6]. This is in contrast to the NF1-P53 and NF1 tumors that maintained a significant decrease in growth compared to the vehicle control. An upward growth trend was observed in the majority of tumors at the 21-day time point, despite ongoing treatment (Figure 1A–C). In the surviving, capmatinib-resistant cells present at 21 days in NF1-MET tumors, distinct changes in the kinome response were observed, comprising consistent AXL (Y702), EGFR (Y1068), cofilin (S3), and AKT (S473) activation (Figure 1D; Figure S5). These results suggest that MET-addicted MPNSTs survive MET inhibition through pathway reactivation via other RTKs (i.e., AXL and EGFR) and potentially a pathway bypass through AKT signaling. In the NF1 tumors whose ascending growth patterns indicated the beginning of drug resistance, increased phosphosite activation was observed in ERK, ribosomal protein S6 kinase (S6), 4EBP1, and AKT, yet markers of parallel RTK activation were also present. In the F1-P53 tumors, which were continuing to grow at the 21-day time point, phosphosite expression returned to levels resembling the vehicle, suggesting that broader kinome adaptation was no longer required for growth. Collectively, these data indicate that distinct mechanisms of innate and adaptive kinome reprogramming occur in genetically diverse MPNSTs.

### 3.2. Kinome Response to MEK Inhibition Results in Bypass Activation

**Figure 2:**
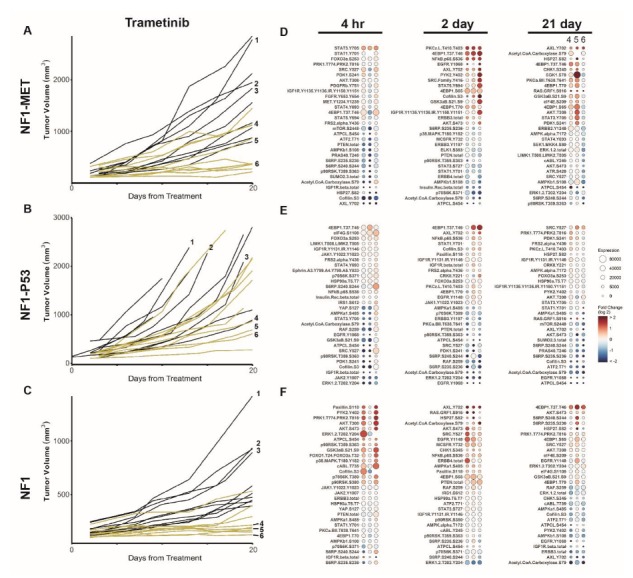
MEK inhibition reveals differential innate and adaptive kinome reprogramming. Individual tumor growth curves for (**A**) NF1-MET, (**B**) NF1-P53, and (**C**) NF1 tumorgrafts plotted by treatment (colored lines) compared to the vehicle (black lines). The analysis of tumor growth data was previously reported [6]. The annotated tumors were analyzed by RPPA (**D**–**F**). The fold change relative to the mean protein expression of control tumors (i.e., #1–3) was calculated for each tumor #4–6, with the first column of Panel A at 21 days corresponding to tumor #4, the second to tumor #5, and the third to tumor #6. Ranked balloon plots of the proteins with the highest and lowest fold change in expression after 4-h, 2-day, and 21-day treatment of the NF1-MET model with trametinib. Each column represents a single animal. Balloon color indicates fold change in expression relative to the vehicle mean (*n* = 3) for that time point. Balloon size indicates the absolute protein expression normalized to the total protein input and background.

The recent clinical success of MEK inhibition with selumetinib in NF1 plexiform neurofibromas and recent preclinical MPNST treatment studies highlight the therapeutic potential of targeting MEK in NF1-related MPNSTs [28,29,30]. To evaluate the kinome response to MEK inhibition in NF1-deficient MPNSTs with distinctive genomic backgrounds, we used the MEK inhibitor trametinib (Novartis, Cambridge, MA, USA). Trametinib is a reversible, highly selective, allosteric inhibitor of MEK1 and MEK2, which is FDA approved for melanoma, lung cancer, and anaplastic thyroid cancers with BRAF mutations. MEK inhibition significantly decreased tumor growth in all of the MPNST lines, yet substantial response heterogeneity was observed in the NF1-MET and NF1-P53 tumors (Figure 2A–C) [6]. The most uniform tumor inhibition was observed in the NF1 MPNST tumors, whereas some NF1-P53 tumors still displayed aggressive growth after 21 days. As with capmatinib, RAS and AKT pathway inactivation (i.e., ERK1/2, mTOR, S6, and p90RSK) was observed after 4 h of trametinib in the NF1-MET and NF1-P53 tumors (Figure 2D,E). Interestingly, broader kinome activation was not observed in these same genomic contexts, suggesting that NF1-MET and NF1-P53 tumors maintain a limited MEK dependency due to innate resistance (Figure 2A,B,D,E; Figure S2). Interestingly, by 2 days, trametinib treatment induced a similar response to capmatinib in the NF1-MET tumors, strongly activating EGFR (Y1068), AXL (Y702), PKCz (L410/T403), and NFκB (S536) (Figure 2D; Figure S5). In contrast, trametinib treatment resulted in the differential regulation of EGFR (Y1068) and AXL (702) in NF1-P53 tumors, as AXL (702) was upregulated, while EGFR (Y1068) was the most repressed site after 2 days (Figure 2D,E). AXL (Y07) was also highly induced after 2 days of trametinib treatment in the NF1 tumors (Figure 2F), suggesting that AXL activation may be a universal early response to MEK inhibition, regardless of the genetic context of the MPNST.

Adaptive kinome reprogramming in response to trametinib was distinct for each model. In the NF1-MET tumors, AXL (Y702) remained activated after 21 days of treatment. 4EBP1 (T37/T46), CHK1 (S345), and AKT (e.g., SGK and AKT) phosphorylation was observed in response to long-term MEK inhibition (Figure 2D; Figure S5). These results implicate a bypass mechanism of resistance to MEK inhibition, particularly in disparate signaling nodes within the AKT and mTOR pathways (i.e., SGK, CHK1, AKT, 4EBP1, and HSP27). Notably, after 21 days of treatment, 90% of NF1-P53 tumors had increasing growth trends and a negligible kinome response to MEK inhibition (Figure 2B,E). ERK was consistently inhibited by trametinib in these resistant tumors, confirming that ERK pathway reactivation was not required to maintain growth. In the NF1 tumors, AKT/mTOR and protein translation pathway effectors were the strongest targets activated by MEK inhibition. Collectively, the NF1-MET kinome response to both MET and MEK inhibition suggests that RTK-dependent MPNSTs may survive kinase inhibition both by the engagement of alternative RTKs (i.e., AXL and EGFR) and increasing AKT/mTOR signaling pathways.

### 3.3. Kinome Response to Combined MET and MEK Inhibition in NF1-Related MPNSTs

**Figure 3:**
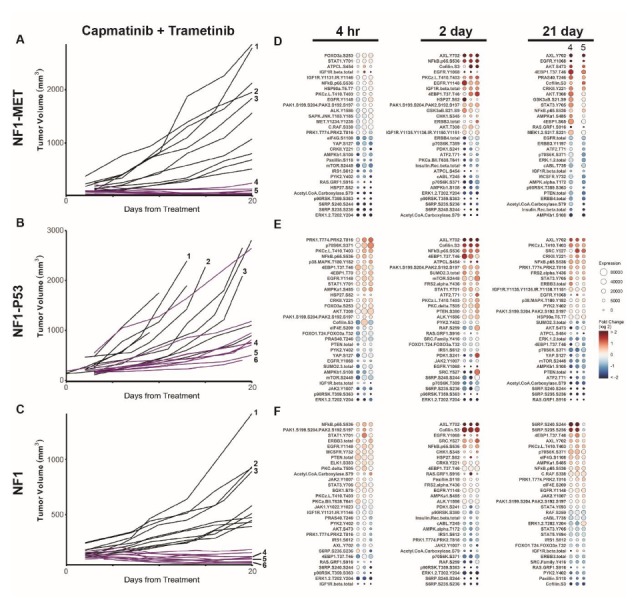
Combination MEK and MET inhibition reveals differential innate and adaptive kinome reprogramming. Individual tumor growth curves for (**A**) NF1-MET, (**B**) NF1-P53, and (**C**) NF1 tumorgrafts plotted by treatment (colored lines) compared to the vehicle (black lines). The analysis of tumor growth data was previously reported [6]. The annotated tumors were analyzed by RPPA (**D**–**F**). The fold change relative to the mean protein expression of control tumors (i.e., #1–3) was calculated for each tumor #4–6, with the first column of Panel A at 21 days corresponding to tumor #4, the second to tumor #5, and the third to tumor #6. Ranked balloon plots of the proteins with the highest and lowest fold change in expression after 4-h, 2-day, and 21-day treatment of the NF1-MET model with combination therapy. Each column represents a single animal. Balloon color indicates the fold change in expression relative to the vehicle mean (*n* = 3) for that time point. Balloon size indicates the absolute protein expression normalized to the total protein input and background.

Since we observed both pathway reactivation and bypass resistance mechanisms with single-agent MET or MEK inhibition, we sought to determine whether targeting multiple signaling pathways may abrogate these kinome adaptations and achieve a more durable clinical response. Previously, we compared combined MET and MEK inhibition with monotherapy and demonstrated significant improvement in tumor inhibition and response variability with combination therapy compared to a single agent alone (Figure 3A–C) [6]. Even in NF1-P53 tumors which had the most heterogeneous responses to monotherapy with capmatinib or trametinib, we observed stable disease in all but one tumor (Figure 3B). The kinome response to combined MET-MEK inhibition exhibited striking differences in comparison to single kinase inhibition. At 4 h and 2 days of treatment, ERK1/2, S6 (S240/S244 and S235/S236), and p90RSK demonstrated the strongest decrease in phosphorylation in all of the MPNST models, suggesting that the RAS/ERK and AKT/mTOR pathways are robustly inactivated with combined MET-MEK inhibition (Figure 3D–F). As with single kinase inhibition, we observed NFκB/p65 (S536) activation at the 2-day time point. We also measured an increase in PKCζ/λ (T410/T4033) and cofilin (S3) phosphorylation at the 2-day time point with both single and combined kinase inhibition (Figure S5). Cofilin is an actin depolymerizing factor known to regulate actin dynamics and cell invasion; however, recent studies have established the role of cofilin in NFκB nuclear translocation [31,32]. The atypical protein kinase C member PKCζ is involved in several survival pathways that are deregulated in cancer and is also involved in the activation of NFκB [33,34]. Together, these findings indicate that NFκB activation is an acute response that occurs in response to monotherapy or combined kinase inhibition in MPNSTs.

At 21 days, significant tumor inhibition was observed in NF1-MET tumors and in the surviving cells, the kinome adaptations observed with single MET inhibition were intensified (Figure 3D; Figure S3A). Specifically, AXL (Y702), EGFR (Y1068), and AKT (S473) are strongly activated. Intriguingly, combined MET-MEK inhibition also resulted in the strong activation of AXL (Y702) in the NF1-P53 and NF1 tumors, which was not observed with single-agent treatment of either drug (Figure 3E,F). The NF1-P53 tumors maintained an inflammatory kinome response after 21-day treatment (PKCζ/λ, NFκB), which stands in contrast to the NF1-MET and NF1 tumors, where an inflammatory response was only observed at 4 h and 2 days (Figure 3D–F; Figure S5). Rather, after 21 days of combination therapy, the surviving cells of NF1 tumors robustly activated S6 (S240/S244 and S235/S236) and 4EBP1 (T37/T46), along with AXL (Y702) (Figure 3F). Given that AXL is activated in response to MET and MEK inhibition in all three of these genomically diverse MPNST models, AXL activation may be a common mechanism of therapeutic resistance to RAS pathway inhibitors.

### 3.4. Kinome Response to Doxorubicin in NF1-Related MPNSTs

**Figure 4:**
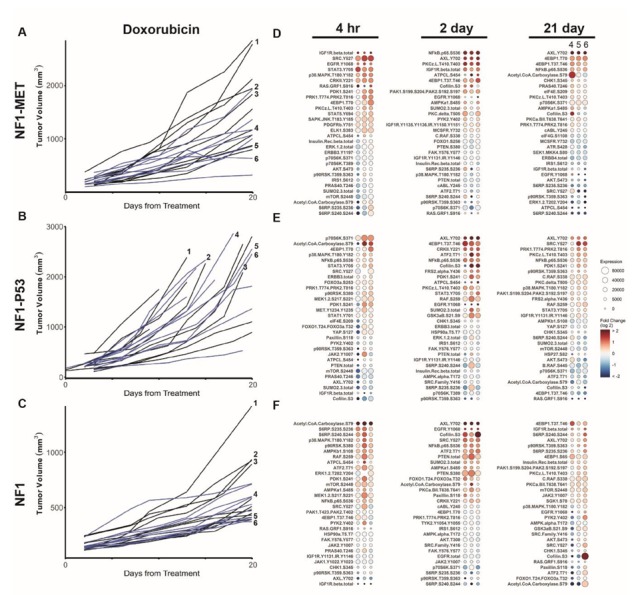
Doxorubicin reveals differential innate and adaptive kinome reprogramming. Individual tumor growth curves for (**A**) NF1-MET, (**B**) NF1-P53, and (**C**) NF1 tumorgrafts plotted by treatment (colored lines) compared to the vehicle (black lines). The annotated tumors were analyzed by RPPA (**D**–**F**). The fold change relative to the mean protein expression of control tumors (i.e., #1–3) was calculated for each tumor #4–6, with the first column of Panel A at 21 days corresponding to tumor #4, the second to tumor #5, and the third to tumor #6. Ranked balloon plots of the proteins with the highest and lowest fold change in expression after 4-h, 2-day, and 21-day treatment of the NF1-MET model with doxorubicin. Each column represents a single animal. Balloon color indicates the fold change in expression relative to the vehicle mean (*n* = 3) for that time point. Balloon size indicates the absolute protein expression normalized to the total protein input and background.

Doxorubicin is a topoisomerase II inhibitor that prevents cellular replication by indirectly stabilizing double-stranded DNA breaks [35]. It has also been implicated in direct DNA damage through free radical production. Doxorubicin is currently being tested in combination with multiple kinase inhibitors for sarcoma (e.g., PDGFα inhibitor), or to treat anthracycline-resistant sarcomas [36,37,38]. Although the results of these trials are mixed, it is unclear whether doxorubicin resistance is mediated at least in part through kinome adaptation. How NF1-related MPNSTs confer doxorubicin resistance is likely multifactorial [39]; however, the patterns of compensatory kinase signaling have not been studied to date. Following the doxorubicin treatment of NF1-MET, NF1-P53, and NF1 tumorgrafts, significant resistance and response heterogeneity was observed (Figure 4A–C). NF1 tumor growth was significantly slower than controls; however, no tumors ultimately responded to treatment. RPPA analysis revealed a broad and diverse response to doxorubicin at early and late timepoints across all genomic contexts. Early responses at 4 h included RTK activation (EGFR, IGF1R, PDGFR, and MET), pro-inflammatory signaling mediators (p38, NFκB, and STAT3/5), and upstream kinases (SRC and RAF) (Figure 4D–F; Figure S4). Kinome responses at 2 days and 21 days of treatment were more diverse, with the emergence of increases in AXL (Y702), EGFR (Y1068), and cofilin (S3) as dominant signaling mediators (Figure S5). Qualitatively, doxorubicin resulted in the broadest pathway responses compared to single-agent capmatinib (Figure 1), trametinib (Figure 2), and combination therapy (Figure 3). Interestingly, the PI3K/AKT/mTOR pathway response did not appear to be significantly activated in response to doxorubicin, as evidenced by the inactivation of AKT (S473), S6RP, and CHK1 (Figure 4D–F, Figure S4). These results indicate that doxorubicin treatment causes both acute and persistent kinome changes in several pathways. The diversity and perseverance of the doxorubicin-mediated kinome response may underlie innate resistance observed in sarcomas.

### 3.5. Combined MET-MEK Inhibition with Doxorubicin Decreases Response Heterogeneity

**Figure 5:**
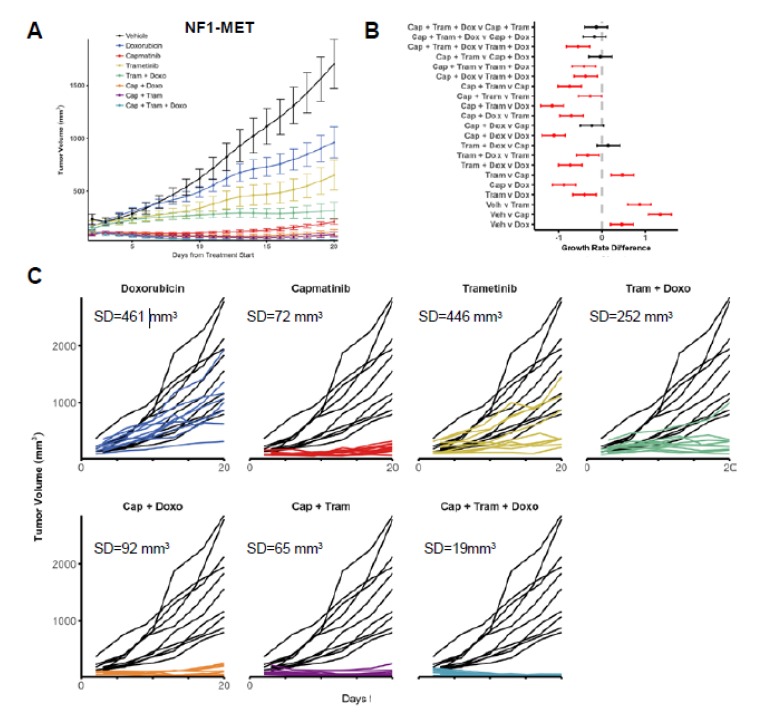

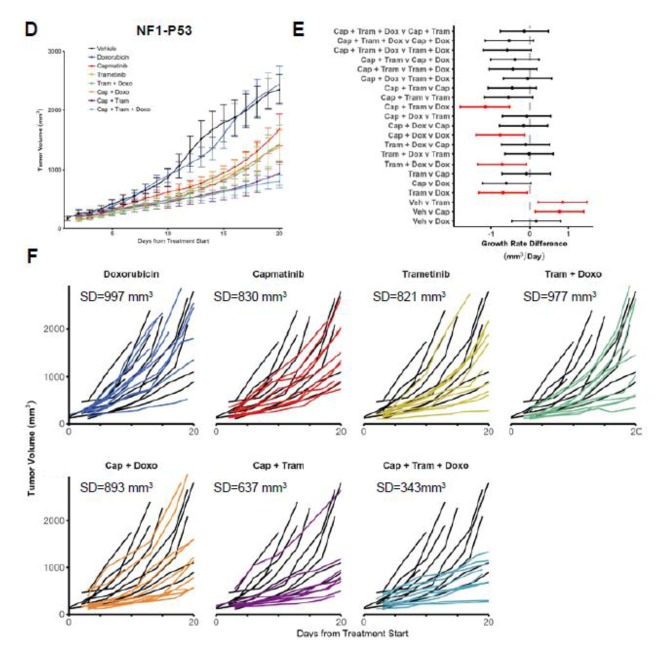
Combined doxorubicin, MET, and MEK inhibitor treatment reduces the response heterogeneity. Tumor growth of (**A**) NF-MET and (**D**) tumorgrafts are plotted as means with standard errors. 95% confidence intervals for the pairwise differences between the growth rates of the select treatments in the (**B**) NF1-MET and (**E**) NF1-P53 tumors, estimated and tested using linear mixed-effects models with random slopes and intercepts, and false discovery rate-adjusted contrasts. Statistically significant differences (*p*-value < 0.05) between compared therapies are highlighted in red. Individual tumor growth curves for (**C**) NF1-MET and (**F**) NF1-P53 tumorgrafts plotted by treatment (colored lines) compared to the vehicle (black lines). The analysis of tumor growth data and differences in treatment response were previously reported for single-agent treatment of capmatinib and trametinib, and combination treatment of capmatinib + trametinib [6].

Because combined MET and MEK inhibition resulted in an improved treatment response in all MPNST lines, we investigated the efficacy of doxorubicin in combination with MET and/or MEK kinase inhibition. As discussed earlier, the kinase inhibition of MET or MEK resulted in distinct kinome adaptations compared to doxorubicin treatment. We focused our tumor growth analysis on the NF1-MET and NF1-P53 tumors since these two MPNST models had the most aggressive growth and distinctive responses to MET and MEK inhibition. In NF1-MET tumors, doxorubicin treatment caused a significant decrease in tumor growth (Figure 5A; *p* < 0.0005); however, doxorubicin treatment was inferior to capmatinib or trametinib (Figure 5B). Even though the mean growth reduction was significant, the heterogeneous response to doxorubicin was substantial (Figure 5C). Combined doxorubicin and kinase inhibition significantly improved tumor inhibition and reduced tumor heterogeneity. For example, trametinib alone resulted in moderate tumor inhibition in NF1-MET tumors (Figure 5A), yet combined trametinib + doxorubicin significantly improved the response in comparison to single-agent treatment with trametinb or doxorubicin (Figure 5B). Since these MPNST tumors are MET-addicted, capmatinib resulted in impressive tumor regression, yet combined capmatinib + trametinib was superior to capmatinib alone (Figure 5B), whereas capmatinib + doxorubicin did not significantly improve the treatment response. The treatment that resulted in the least response variability (SD = 19 mm) was the capmatinib + trametinib + doxorubicin combination, with each tumor showing consistent growth inhibition.

For the MPNST tumorgraft line, the NF1-P53 tumors had the most aggressive growth, highest response heterogeneity, and least impressive response to single-agent treatment. In NF1-P53 tumors, doxorubicin treatment did not result in tumor regression (Figure 5D–F). Combined doxorubicin and kinase inhibition reduced tumor growth in comparison to doxorubicin alone, yet this combination was not better than capmatinib or trametinib single-agent treatment (Figure 5E). The triple combination of capmatinib + trametinib + doxorubicin was not significantly better than capmatinib + trametinib; however, this treatment combination resulted in the least heterogeneity in the response (Figure 5F; SD = 343 mm^3^). The heterogeneity of response and growth patterns observed correlated with the diversity and intensity of the innate and acquired kinome responses delineated in these genomically distinct MPNST tumors.

### 3.6. ERK Reactivation is Observed in Cells Resistant to MET or MEK Inhibition

**Figure 6:**
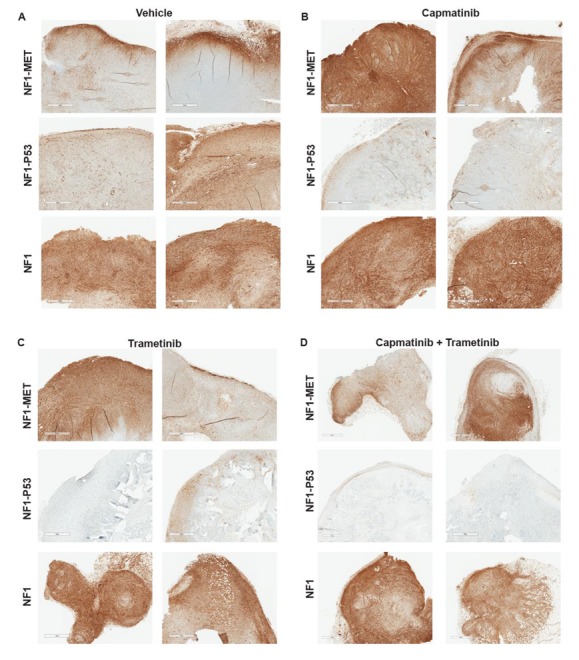

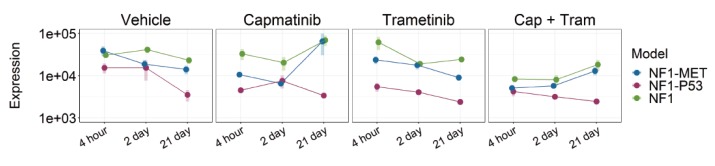
ERK reactivation or pathway indifference drive resistance to kinase inhibition. Phospho-ERK1/2 T202/Y204 expression in each genetic model after 21 days of (**A**) vehicle, (**B**) capmatinib, (**C**) trametinib, or (**D**) combination treatment. (**E**) phospho-ERK1/2 T202/Y204 expression values were measured by RPPA and calculated as the absolute protein expression normalized to the total protein input and background. Points represent the mean (*n* = 3) for each treatment-genotype-time group. Shaded bars represent +/− SEM.

RPPA revealed the consistent de-repression or reactivation of ERK (T202/Y204) in surviving cells throughout 21 days of kinase inhibitor treatment. To determine if ERK reactivation was specific to resistant subpopulations or the entire tumor, we stained the tumors for phospho-ERK (T202/Y204) after 21 days of single or combination therapy. In vehicle-treated tumors, we observed moderate to strong pERK staining; however, distinct ERK activation patterns were observed in each tumorgraft line (Figure 6A). In the NF1-MET vehicle tumors, ERK activation was intense at the invasive edge of the tumor, whereas ERK activation was moderate to strong and uniformly expressed in the NF1-P53 and NF1 tumors (Figure 6A). After 21 days of single-agent treatment with either capmatinib or trametinib, ERK activation was robust in the NF1-MET and NF1 tumors (Figure 6A–C). Interestingly, ERK activation decreased with single-agent treatment in the NF1-P53 tumors, except for minor cell populations at the invasive edge in some tumors (Figure 6B,C). This decrease was even more pronounced in the combined capmatinib-trametinib-treated NF1-P53 tumors, while the capmatinib-trametinib NF1-MET and NF1 tumors maintained high levels of ERK activation (Figure 6D). To directly compare RPPA and IHC and to understand how ERK phosphorylation changed over time in each model, we plotted the normalized absolute protein expression measured by RPPA for ERK (T202/Y204) for each treatment (Figure 6E). Overall, ERK maintained a similar level of activation or increased over time in the NF1-MET and NF1 models. Moreover, long-term capmatinib treatment induced strong ERK activation in these tumors, whereas NF1-P53 tumors consistently maintained lower levels of ERK activation compared to the NF1-MET and NF1 tumors (Figure 6E).

### 3.7. AXL NFkB Co-Activation Associated with Therapy Resistance in MPNSTs

**Figure 7:**
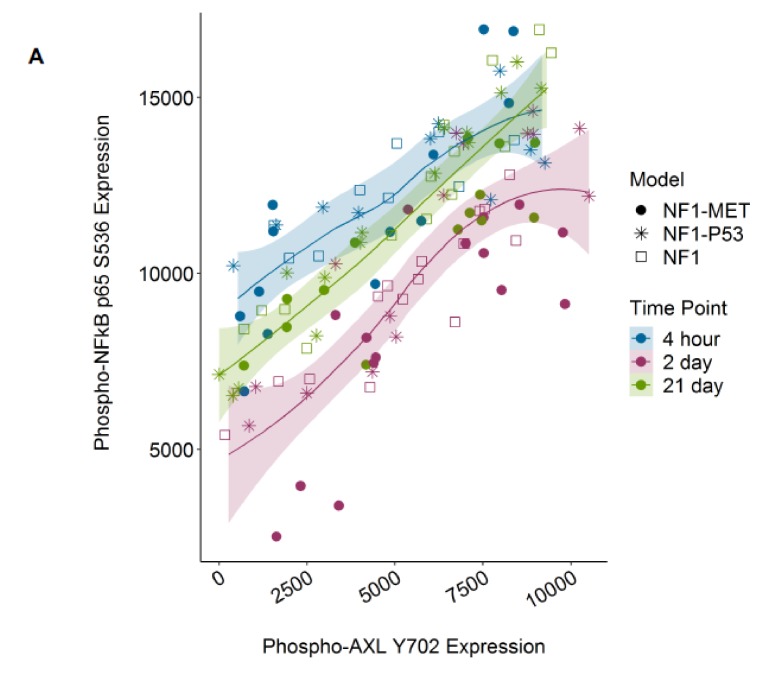

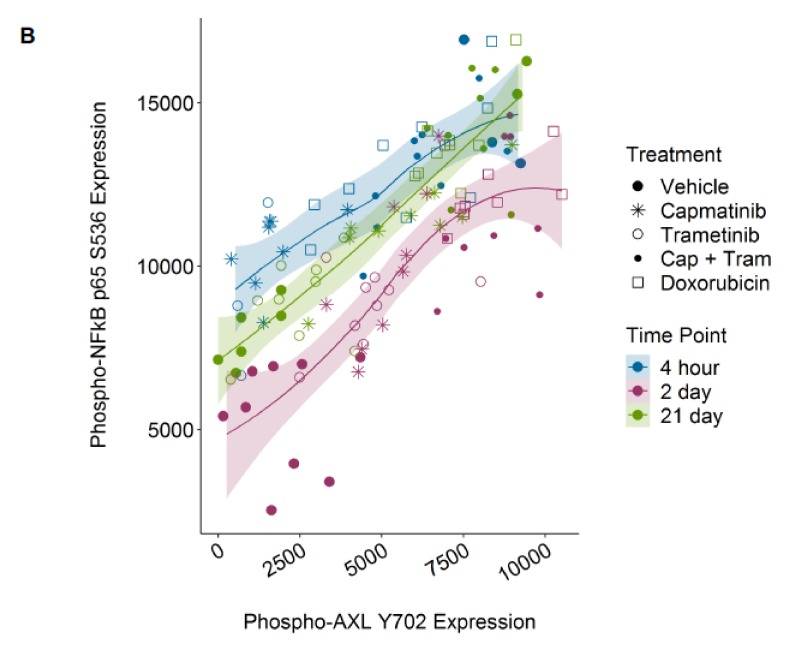
AXL Y702 and NFkB p65 S536 phosphorylation are highly correlated. Expression of phoshpo-AXL Y702 and phospho-NFkB p65 S536 plotted by (**A**) time and genotype group or (**B**) time and treatment group. Colors indicate treatment time and point shape indicates treatment or genotype groups. Lines indicate loess-predicted fit for each time point; shaded regions indicate 95% confidence intervals. Spearmen’s rank correlation rho = 0.832, 0.835, and 0.881 with *p* value = 3.72 × 10^−9^, 9.75 × 10^−13^, and 6.40 × 10^−15^ for the 4-h, 2-day, and-21 day groups, respectively.

As both AXL and NFkB were highly activated in several treatment conditions, including therapy-resistant tumor growth, we sought to determine whether AXL and NFkB phosphorylation were correlated in our models. Recent studies in other cancer contexts suggest that AXL induces NFkB activation in response to a variety of therapies, including kinase inhibitors (22410775, 23474758, and 25568334). This novel therapy mechanism has been underreported to date. We determined that pAXL expression was tightly correlated to pNFkB (Spearmen’s rank correlation rho = 0.729, *p* value = 3.91 × 10^−21^) and grouped strongly by time point (Figure 7A,B). Expression was also grouped by treatment, as the phosphorylation of both proteins was highest in the doxorubin and combination capmatinib + trametinib treatment groups (Figure 7B), regardless of genotype (Figure 7A).

## 4. Discussion

Currently, there is no effective chemotherapy for NF1-related MPNSTs. Despite ongoing clinical trialing efforts, neither RTK nor downstream kinase inhibition has resulted in meaningful improvements in survival, despite well-founded attempts to target both the RAS/ERK and PI3K/AKT/mTOR pathways. Fundamentally, RAS deregulation as a result of *NF1* deficiency appears to be more difficult to target than constitutive RAS activation. One possible reason for this difference is that fewer discrete signaling dependencies exist with *NF1* tumor suppressor loss than cancers that are critically dependent on RAS signaling as a result of activating RAS or EGFR mutations. That is to say, NF1-related MPNSTs are less susceptible to the perturbation of oncogenic signaling, unless a genomic event such as *MET* or *HGF* amplification exhausts the negative feedback loops that drive kinome adaptation. Our data confirms substantially broad and redundant kinome adaptation in NF1-related MPNSTs in response to MET or MEK inhibition. What is even more impressive is the diversity of early and late response mediators which sit atop prominent signaling cascades that regulate growth, proliferation, inflammation, and apoptosis. These data strongly point to an evolutionary advantage in clonal selection for cell populations that maintain this degree of signaling plasticity. Based on our findings, any successful treatment strategy that relies solely on kinase inhibition will be difficult to sustain. Even in our most ideal treatment scenario where the potent MET inhibitor capmatinib suppressed growth in MET-addicted MPNSTs, kinome adaptation occurred within 21 days, leading to a slight resumption in growth by the end of the study time frame.

Proteomic profiling analysis provided significant insight into how the genomic context influenced the therapy response, particularly in the case of oncogene addiction. The “MET-addicted” NF1-MET tumor model is highly responsive to MET inhibition. We confirmed this finding with proteomic profiling by showing a sharp decrease in MET activation and downstream signaling immediately after capmatinib treatment. Even though MET inhibition was sustained at 2 days, strong AXL and AKT activation indicated the initiation of pathway reactivation and pathway bypass signaling. After 21 days of capmatinib treatment, resistant populations reprogrammed the kinome via AXL and EGFR. Besides activation of the AKT/mTOR pathway, ERK reactivation was consistently present in NF1-MET tumors, even in the minor cell populations that survived combined MET-MEK inhibition. In contrast to the MET-addicted tumors, few, if any, signaling dependencies were present in the capmatinib-treated NF1-P53 tumorgrafts. Interestingly, the pace and strength of kinome adaptations in NF1-P53 tumors were considerably reduced compared to NF1-MET and NF1 tumors. One possible reason for this observation is that MET inhibition failed to cause sufficient cellular stress to necessitate broad kinome adaptation in innately-resistant NF1-P53 tumors. MEK inhibition was confounded by a higher degree of innate resistance than MET inhibition and resulted in greater response heterogeneity and variability in kinome activation within genotype groups. As observed with MET inhibition in NF1-P53 tumors, less robust kinome activation was observed in response to trametinib in NF1-P53 tumors.

In both capmatinib- and trametinib-treated tumors, inflammatory signaling was present at the 4-h time point, whereas kinase signaling associated with proliferation and invasion dominated at 2 days and 21 days. Inflammation has not been widely studied in MPNSTs; however, it is a key determinant of neurofibroma progression and Schwann cell homeostasis [40,41,42]. Based on our data, all treatments were associated with an initial inflammatory response directly mediated by the kinome. Multiple targets were implicated, including key members of the JAK/STAT signaling cascade; however, NFkB was the most consistently activated target. NFkB activation results in pleiotropic effects, including broad transcriptional activation, cytokine production, and cell survival. It is an early response element to cellular stress with a known ability to activate multiple kinases. How NFkB contributes to kinome adaptation is currently unknown. These results suggest that further investigations into the inflammatory signaling and the impact of the tumor microenvironment may identify additional therapeutic targets for NF1-related MPNSTs.

AXL receptor activation was a consistent kinome adaptation observed in all of the MPNST models in response to kinase inhibition and doxorubicin treatment. Recently, AXL has been implicated in therapy resistance to multiple targeted therapies and cancer types, including MPNST. Resistance is often mediated through AXL dimerization with other RTKs, leading to the bypass of the RTK inhibitor effect [43,44]. For example, in ovarian tumors, AXL dimerizes with MET, EGFR, and HER2, leading to sustained ERK activation [45]. In response to ERK and MEK inhibition, AXL/MITF-mediated drug resistance is observed among mutant BRAF and NRAS melanoma cell lines [46]. AXL overexpression has also been observed in resistance to cytotoxic chemotherapies, such as docetaxel, in prostate cancer [47]. A principal role of AXL appears to be sustaining a mesenchymal phenotype, which is a mechanism of resistance to diverse anticancer therapies [43]. We demonstrated that AXL and NFkB activation are highly correlated, regardless of the treatment type or model genotype. These results strongly point to a unifying mechanism of therapy resistance in NF1-related MPNSTs. Therefore, further investigations into the efficacy of AXL or NFkB inhibition in conjunction with RAS pathway inhibitors in MPNSTs are warranted.

In summary, the phosphoproteomic profiling of MET and MEK inhibition revealed distinct pathways of drug resistance involving AXL activation, ERK reactivation, and inflammatory kinase signaling. As expected from our previous studies, P53-null MPNSTs were innately resistant to kinase inhibition and demonstrated the most heterogeneous kinome responses; however, combined MET-MEK inhibition exposed potential vulnerabilities in these tumors. As with other RAS-activated tumors, pathway reactivation and bypass signaling are common mechanisms of therapeutic resistance. The results in this study point toward specific vulnerable signaling nodes in MPNSTs that may be exploited through novel combination therapeutic approaches.

## Supplementary Materials

The following are available online at https://www.mdpi.com/2073-4425/11/3/331/s1, Figure S1: MET inhibition reveals differential innate and adaptive kinome reprogramming. Figure S2: MEK inhibition reveals differential innate and adaptive kinome reprogramming. Figure S3: Combination MEK and MET inhibition reveals differential innate and adaptive kinome reprogramming. Figure S4: Doxorubicin reveals differential innate and adaptive kinome reprogramming. Figure S5: AXL, PKCζ/λ, p38, and NFkB phosphorylation with treatment over time.
