# Supplementary material for: Kinome Profiling of NF1-Related MPNSTs in Response to Kinase Inhibition and Doxorubicin Reveals Therapeutic Vulnerabilities
Source: Genes (Basel). 2020 Mar 20;11(3):331. doi: 10.3390/genes11030331 (PMC7141129; doi:10.3390/genes11030331)
Supplement: Supplementary file 1 [file genes-11-00331-s001.zip › Supp_Figure_1_Final.pdf]

Supplemental Figure 1

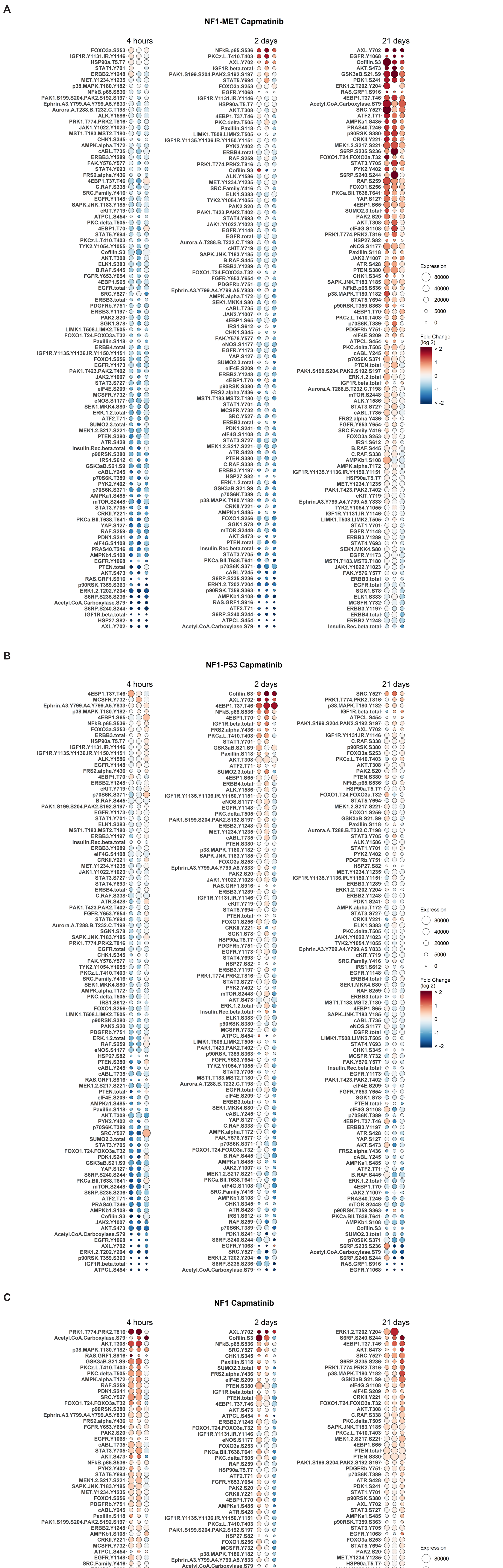

Supplemental Figure 1. MET inhibition reveals differential innate and adaptive kinome reprogramming. Ranked balloon plots of capmatinib treated (A) NF1-MET, (B) NF1-P53, and (C) NF1 tumors after 4 hours, 2 days, and 21 days of treatment. Each column represents a single animal. Balloon color indicates fold change in expression relative to the vehicle mean (n=3) for that time point. Balloon size indicates absolute protein expression normalized to total protein input and background.
