# Supplementary material for: Kinome Profiling of NF1-Related MPNSTs in Response to Kinase Inhibition and Doxorubicin Reveals Therapeutic Vulnerabilities
Source: Genes (Basel). 2020 Mar 20;11(3):331. doi: 10.3390/genes11030331 (PMC7141129; doi:10.3390/genes11030331)
Supplement: Supplementary file 1 [file genes-11-00331-s001.zip › Supp_Figure_5_gene_specific_final.pdf]

Supplementary Figure 5

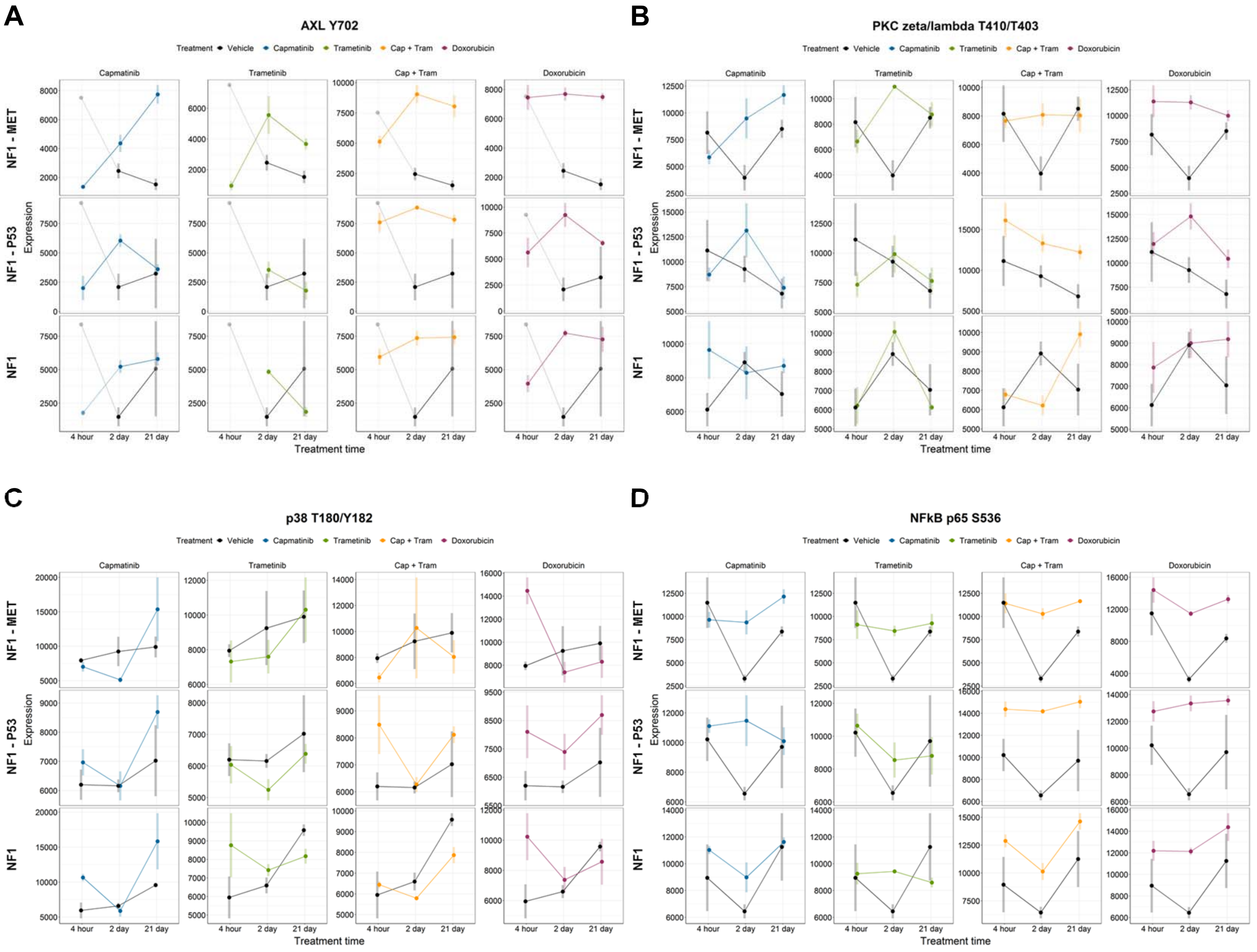

Supplemental Figure 5. AXL, PKCζ/λ , p38, and NFkB. Phospho- (A) AXL(Y702), (B) PKCζ/λ(T403/T410), (C) p38(T180/T182), (D) NFkBp65(S536) expression values measured by RPPA and calculated as absolute protein expression normalized to total protein input and background. Points represent the mean (n=3) for each treatment-genotype-time group. Shaded bars represent +/-SEM.
