# Supplementary figures and images for: Kinome Profiling of NF1-Related MPNSTs in Response to Kinase Inhibition and Doxorubicin Reveals Therapeutic Vulnerabilities
Source: Genes (Basel). 2020 Mar 20;11(3):331. doi: 10.3390/genes11030331 (PMC7141129; doi:10.3390/genes11030331)

## Supplemental Figure 4

**A**

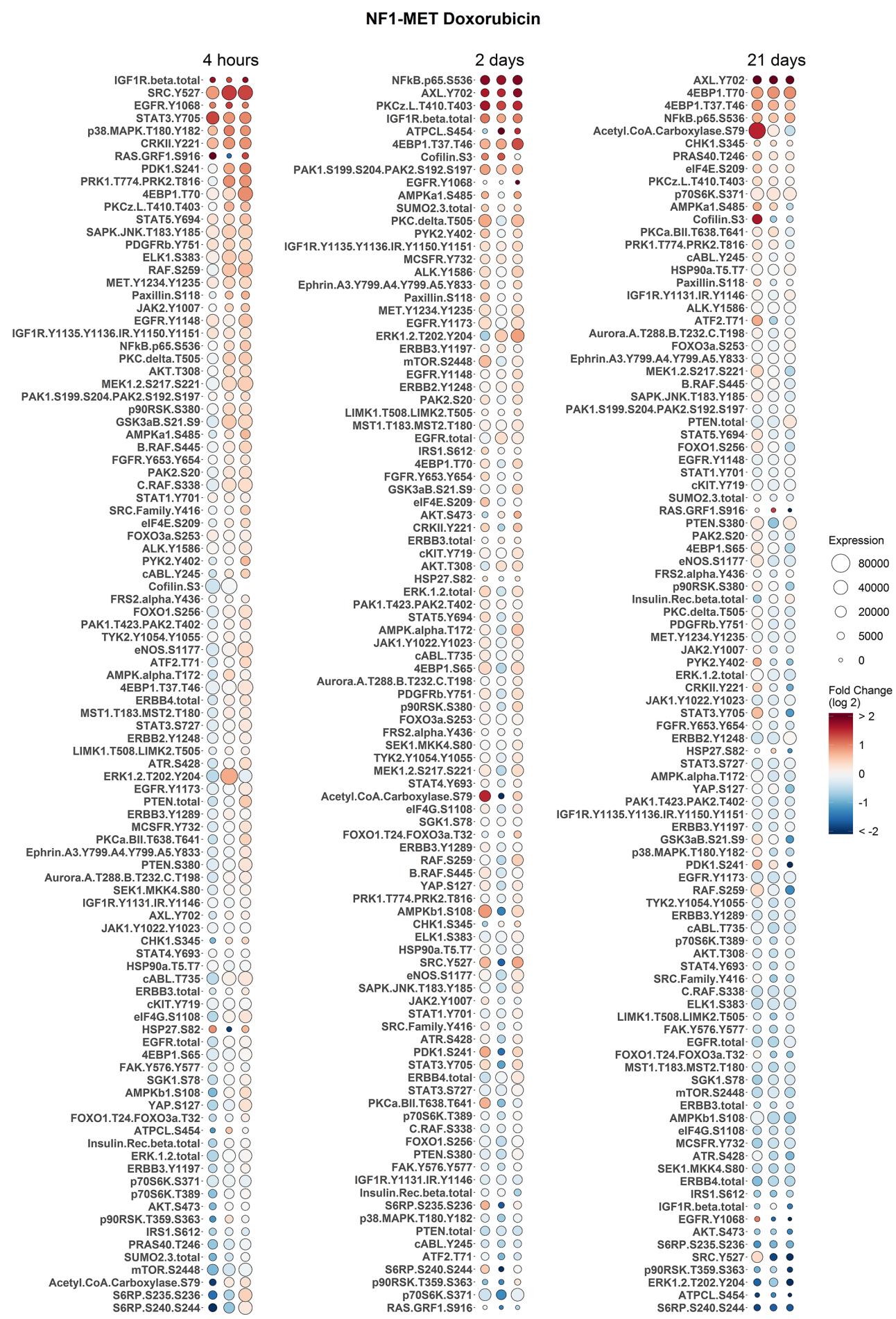

**B**

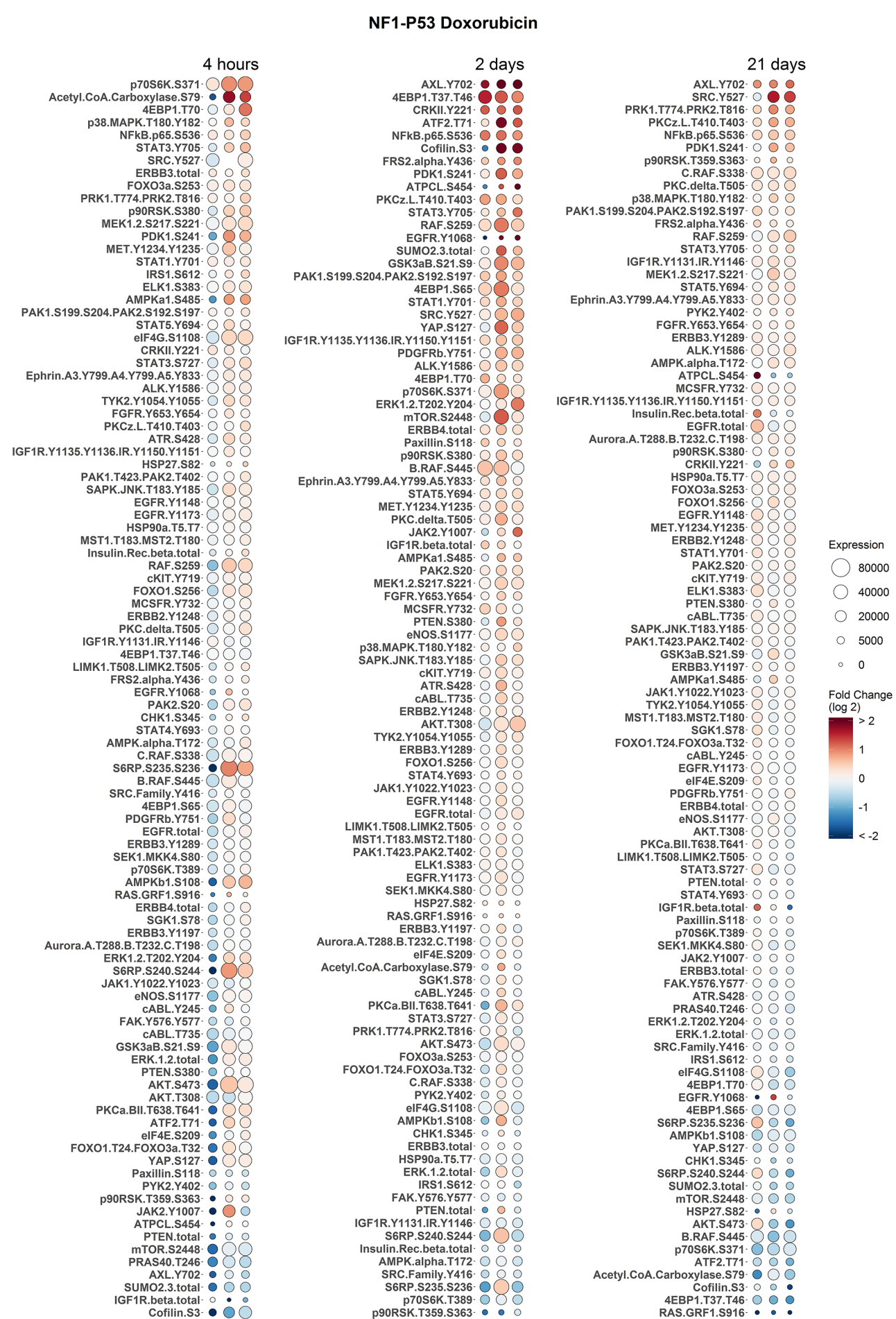

C

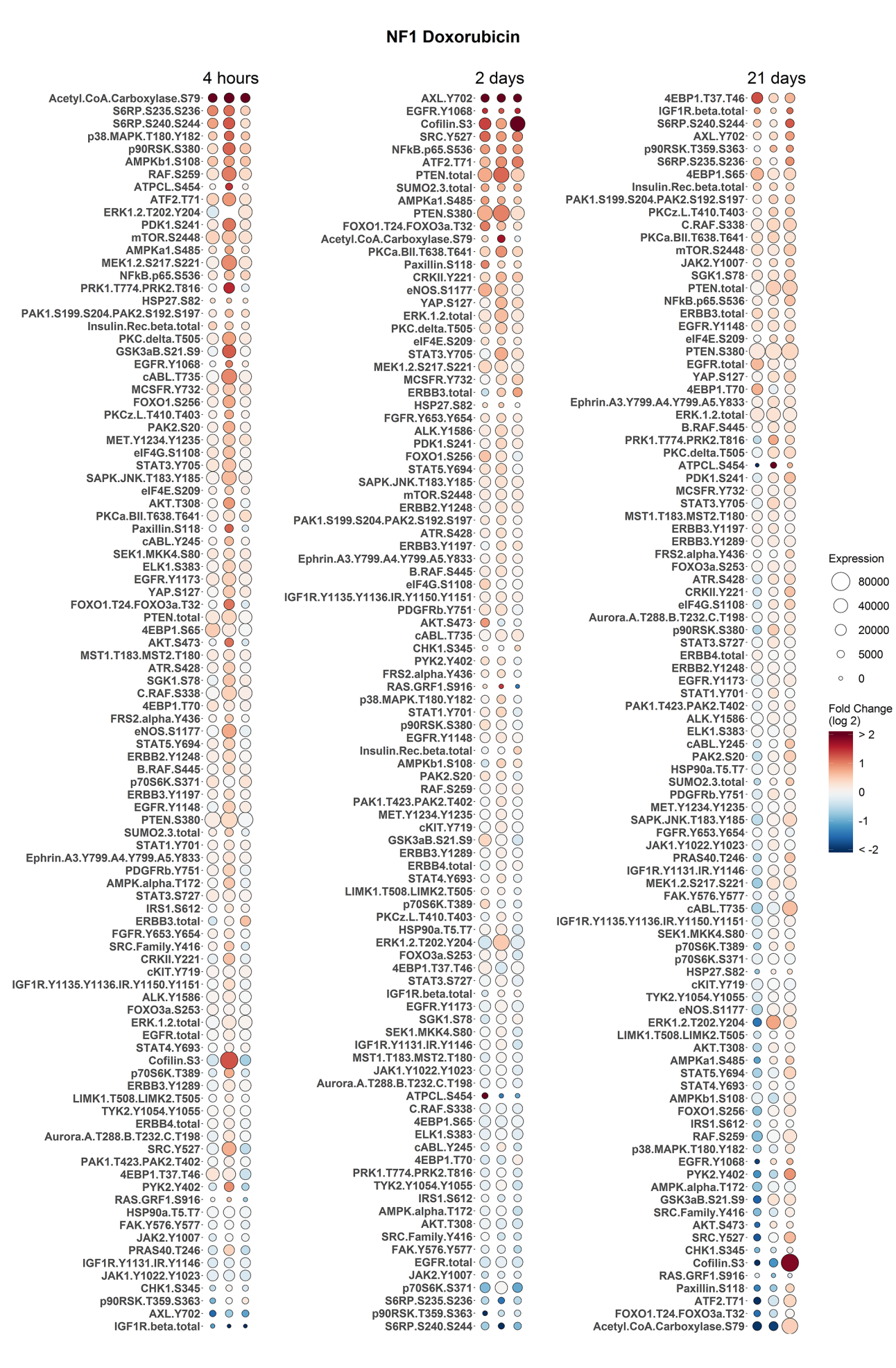

Supplement: Supplementary file 1 [file genes-11-00331-s001.zip › Supp_Figure_4_Final.pdf]
